# A multi-task learning model for predicting drugs combination synergy by analyzing drug–drug interactions and integrated multi-view graph data

**DOI:** 10.1038/s41598-023-48991-9

**Published:** 2023-12-18

**Authors:** Samar Monem, Aboul Ella Hassanien, Alaa H. Abdel-Hamid

**Affiliations:** 1 Mathematics and Computer Science Department, Faculty of Science, Beni-Suef University, Beni-Suef, 62521 Egypt; 2 Faculty of Computer and AI, Cairo University, Cairo, Egypt; 3 Scientific Research Group in Egypt (SRGE), ,

**Keywords:** Cancer, Computational biology and bioinformatics, Drug discovery

## Abstract

This paper proposes a multi-task deep learning model for determining drug combination synergistic by simultaneously output synergy scores and synergy class labels. Initially, the two drugs are represented using a Simplified Molecular-Input Line-Entry (SMILE) system. Chemical structural features of the drugs are extracted from the SMILE using the RedKit package. Additionally, an improved Multi-view representation is proposed to extract graph-based drug features. Furthermore, the cancer cell line is represented by gene expression. Then, a three fully connected layers are learned to extract cancer cell line features. To investigate the impact of drug interactions on cell lines, the drug interaction features are extracted from a pretrained drugs interaction network and fed into an attention mechanism along with the cancer cell line features, resulting in the output of affected cancer cell line features. Subsequently, the drug and cell line features are concatenated and fed into an attention mechanism, which produces a two-feature representation for the two predicted tasks. The relationship between the two tasks is learned using the cross-stitch algorithm. Finally, each task feature is inputted into a fully connected subnetwork to predict the synergy score and synergy label. The proposed model ‘MutliSyn’ is evaluated using the O'Neil cancer dataset, comprising 38 unique drugs combined to form 22,737 drug combination pairs, tested on 39 cancer cell lines. For the synergy score, the model achieves a mean square error (MSE) of 219.14, a root mean square error (RMSE) of 14.75, and a Pearson score of 0.76. Regarding the synergy class label, the model achieves an area under the ROC curve (ROC-AUC) of 0.95, an area under the precision-recall curve (PR-AUC) of 0.85, precision of 0.93, kappa of 0.61, and accuracy of 0.90.

## Introduction

In complex diseases, the effectiveness of targeting multiple biochemical strategies within cells using a single drug focused on a single target is limited. To overcome this inefficiency, drug combination therapy emerges as a promising treatment approach that combines multiple drugs to achieve superior therapeutic effects beyond what individual drugs can achieve alone. Another advantage is the reduction in side effects by minimizing the required dosage for each drug. The success of drug combination therapy has been evident for several decades, particularly in addressing the common occurrence of drug resistance in cancer. Consequently, identifying optimal drugs combination is a critical task with far-reaching implications for translational, clinical, and financial research. The synergy score plays a crucial role in determining the effectiveness of drug combinations.

Testing the synergy of drug combinations through experiments becomes impractical when dealing with a large number of combinations in high-throughput screens. Experimental approaches are not only risky but also costly and time-consuming, demanding substantial human resources, research experience, and technical expertise. Consequently, deep learning models have emerged as valuable tools in biomedical applications, offering the ability to simulate and analyze biomedical data in a more efficient and scalable manner. So, several approaches have been suggested for constructing a simulation model capable of predicting the target of drug combinations. These techniques rely on various features, including chemical drug features, structural network interactions, and cell line omics data.

Among these techniques, DeepSynergy^1^ was the pioneer in applying deep learning models, surpassing other machine learning algorithms in performance. Subsequently, the MatchMaker technique^2^ improved the structure of the deep learning network. TranSynergy^3^ proposed a novel representation of drugs based on selected genes for drug target genes, employing a transformer network. AuDNNsynergy^4^ leveraged full omics data, including gene expression, copy number, and genetic mutation data, to represent the cell line. DeepDDS^5^ utilized a graph attention network to extract drug features. Lastly, PRODeepSyn^6^ investigated the impact of gene interactions in cancer cell lines. Despite the diverse perspectives employed by these techniques, the prediction of drug combination synergy remains a challenging problem.

In this paper, we present an integrated multi-task model that enables simultaneous prediction of both the synergy score and synergy class label of drug combinations. Our approach incorporates various techniques to effectively represent the drugs and the cell line, enabling comprehensive analysis.

To represent the drugs, the SMILE notation is utilized, which allows to extract two distinct features. Firstly, the Mordred package is employed to extract structural chemical SMILE features. Secondly, an improved multi-view graph embedding technique is proposed to extract a feature vector of drugs which represents as a graph.

On the other hand, the cell line is represented using gene expression data obtained from 875 genes, providing a comprehensive depiction of the cell. Additionally, we integrate drug-drug interaction information to study its influence on cancer cell lines. This is achieved by pretraining a drug-drug interaction network with well-known data. The two drugs are then fed into this network to extract drug interaction features.

Next, the cancer cell line and the drug interaction features are fed into an attention mechanism, which produces enhanced features for the cancer cell line. Subsequently, the drug and cell line features are concatenated into a single feature vector, which is further processed by an attention model. The attention model generates two weighted feature vectors that serve as inputs for predicting the synergy score and synergy class label.

To facilitate knowledge transfer between the two tasks, the cross-stitch algorithm is learned to predict their relationship. Finally, two fully connected subnetworks are employed to generate the output for the synergy score and class label, respectively.

Overall, our proposed multi-task model presents a comprehensive and effective framework for predicting drug combination synergies and outperforms other compared methods.

The subsequent sections of this paper are organized as follows: “Preliminary” presents a comprehensive overview of the fundamental knowledge and methodologies employed in the MutliSyn, specifically focusing on attention and cross-stitch algorithms. “MutliSyn” presents a comprehensive description of the MutliSyn, outlining its key components and functionalities. “Experimental results” covers aspects such as the dataset used, evaluation metrics, model parameters, and experimental results. Lastly, “Conclusion” concludes with a summary of the MutliSyn.

## Preliminary

This section begins by introducing the necessary background knowledge on attention and cross-stitch mechanisms separately.

### Attention mechanism

The attention model^7^ is a powerful framework employed in multi-task models. It utilizes attention gates to analyze the complex relationships among vectors, guiding the learning process for different tasks. By incorporating attention methods, the model dynamically assigns varying weights or importance to specific values within a vector set. This enables the model to prioritize and highlight the most informative vectors while disregarding less significant ones. Additionally, the attention model is capable of identifying significant patterns and relationships by selectively attending to vectors relevant to a particular task.

**Figure 1:**
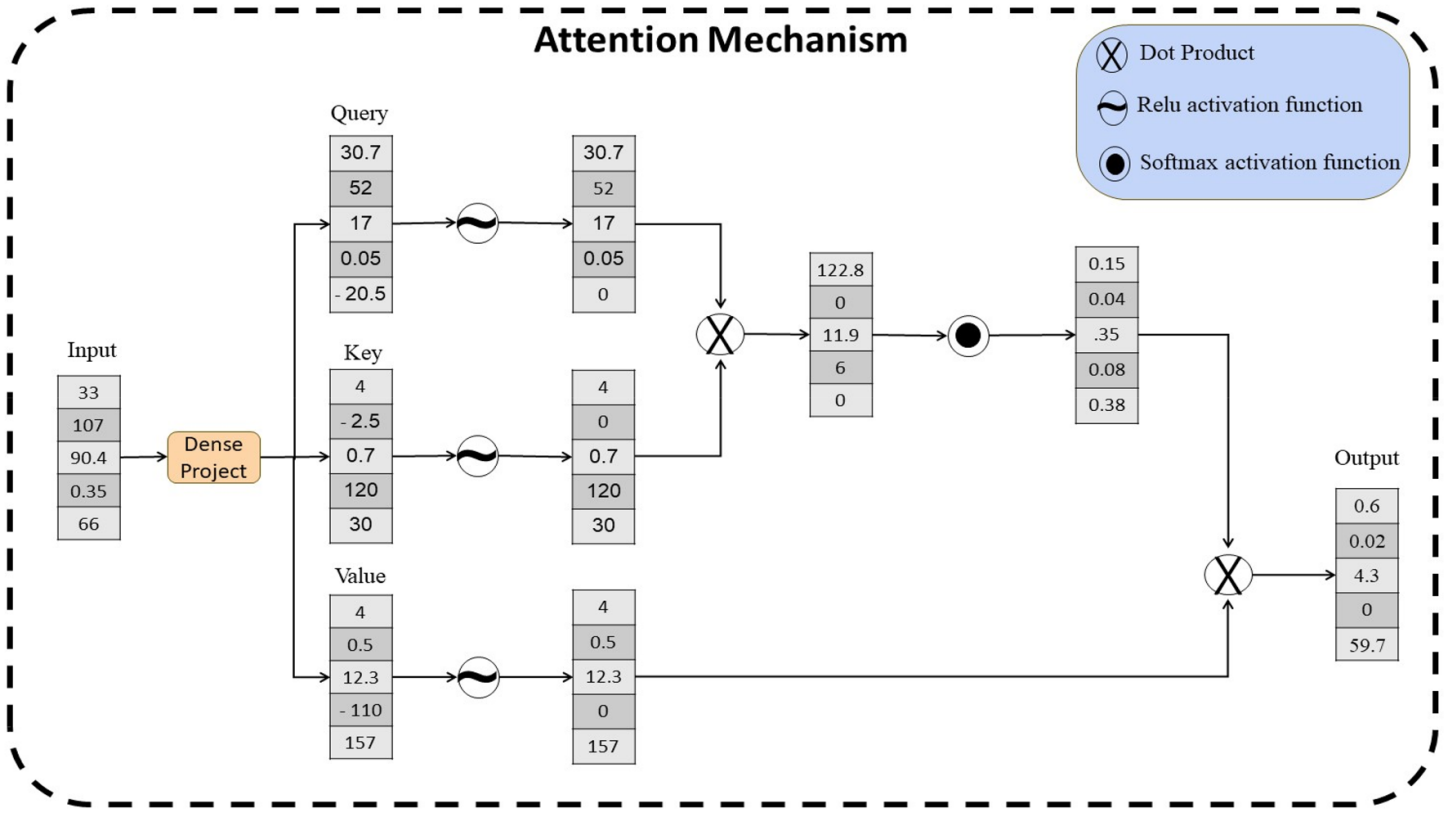
The attention mechanism process that first project the input by using dense layer into query, key, value. Then a dot product is executed between the query and key, accompanied by a softmax activation to generate attention weights. These attention weights are then applied in a dot product operation.

In this research, a multi-head attention approach is employed, enabling separate weighting of extracted features for each task. This mechanism allows the model to effectively navigate through the intricate relationships and dependencies within the data, leading to improved outcomes in multi-task learning and facilitating an exploration of the relationships between tasks. Figure 1 shows the mechanism of multi-head attention.

The multi-attention mechanism involves three primary inputs: query ($q$), key ($k$), and value ($v$) vectors. The attention model maps the input layer ${I}_{0}$ to ${I}_{q}$, ${I}_{k}$, ${I}_{v}$ using separate dense projection layers, as described by Eqs. (1), (2), and (3):

1 $${I}_{q}=f\left(wa*{I}_{0}+b\right)$$

2 $${I}_{k}=f\left(wa*{I}_{0}+b\right)$$

3 $${I}_{v}=f\left(wa*{I}_{0}+b\right)$$

Here, $f$ denotes the activation function ‘relu’ while $wa$ and $b$ denote attention weight and bias vector, respectively.

Subsequently, dot-product attention is employed to these vectors as shown in Eq. (4).

4 $$s=softmax({I}_{q}{*I}_{k})$$

Then, the output is summarized using Eq. (5):

5 $${I}_{f}^{1}=\sum s*{I}_{v}$$

The aforementioned steps are iterated $h$ parallel heads times and the resulting vectors are concatenated together to obtain the final vector according to Eq. (6).

6 $${I}_{f}=concat({I}_{f}^{1},{I}_{f}^{2},\dots \dots ,{I}_{f}^{h}).$$

In this paper, to capture a broader range of information and mitigate potential overfitting issues, the output of the multi-head attention is merged with the input of the multi-head by applying the concatenation operation as specified in Eq. (7).

7 $${I}_{final}=concat({I}_{f},{I}_{0}).$$

### Cross-stitch mechanism

In multi-task models, accurately determining the relationships between tasks is paramount for optimizing performance. By establishing and leveraging these relationships, the model can facilitate the transfer of valuable knowledge from one task to another, thereby enhancing learning and generalization capabilities. This enables more efficient utilization of the available data and facilitates exploration of the interconnections between tasks. As a result, the model can achieve improved performance and accelerated learning in multi-task settings. In contrast, an erroneous configuration of task relationships can have detrimental effects, such as impeding knowledge transfer and diminishing prediction performance. Therefore, this paper employs the cross-stitch algorithm^8^ to explore the relationships between multi-tasks. By leveraging the capabilities of the cross-stitch subnetwork, the model can effectively uncover and establish the appropriate relationships between the tasks, promoting effective knowledge transfer and enhancing prediction performance.

The cross stitch relies on the cross-stitch block to decide how much sharing is needed. The cross-stitch operation is defined in Eq. (8).

8 $$\left[{\overline{t} }_{1} {\overline{t} }_{2}\right]=\left[\begin{array}{cc}{r}_{11}& {r}_{12}\\ {r}_{21}& {r}_{22}\end{array}\right] [{t}_{1} {t}_{2}]$$

Here, ${t}_{1}, {t}_{2}$ represent the input tasks representation, while ${\overline{t} }_{1}, {\overline{t} }_{2}$ denote the output task relationships representation for the respective tasks. The values of ${r}_{ij}$ indicate the learned relationships between tasks $i$ and $j$.

The cross-stitch layer can be summarized as shown in Eq. (9).

9 $$( {\overline{t} }_{1}, {\overline{t} }_{2})=cross\_stitch({t}_{1}, {t}_{2})$$

In this paper, output vectors from cross stitch are separately passed through fully connected layers to generate new tasks representations, ${t}_{11}, {and t}_{22}$, respectively.

Following that, another cross-stitch layer is applied, as depicted in Eq. (10).

10 $$( {\overline{t} }_{11}, {\overline{t} }_{22})=cross\_stitch({t}_{11}, {t}_{22})$$

**Figure 2:**
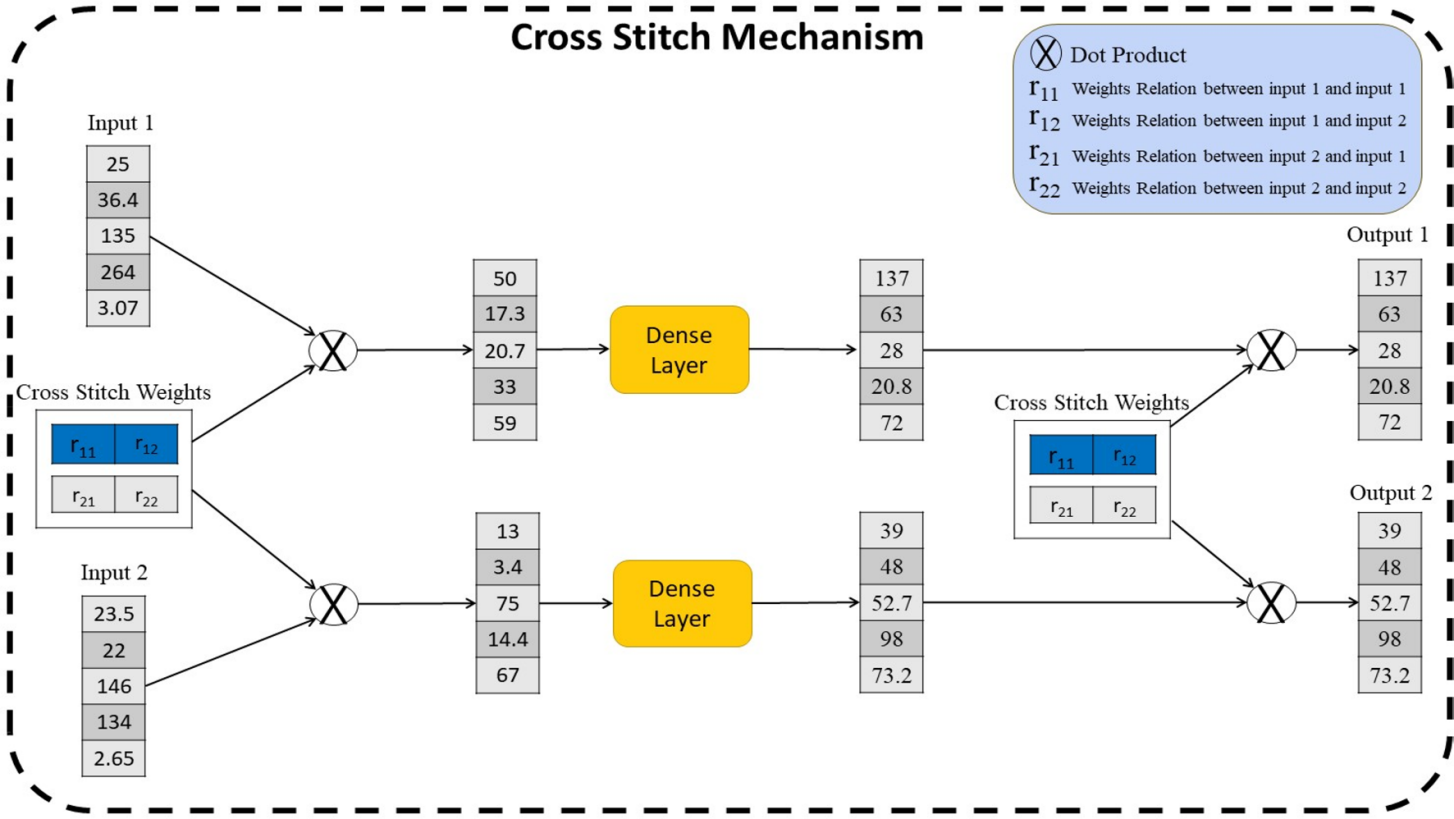
The cross-stitch mechanism process which involves learning relation weights between two tasks. Subsequently, a dot product process is performed between these weights and the corresponding inputs, followed by a dense layer operation. Finally, the output of the dense layer undergoes a dot product with additional cross-stitch weights to yield the learned relation outputs.

Finally, the input and output vectors of the cross-stitch subnetwork are concatenated, as indicated in Eqs. (11) and (12). Figure 2 shows the layers of cross-stitch applied in this paper.

11 $${t}_{final1}=concat({\overline{t} }_{11},{t}_{1})$$

12 $${t}_{final2}=concat({\overline{t} }_{22},{t}_{2})$$

## MutliSyn

**Figure 3:**
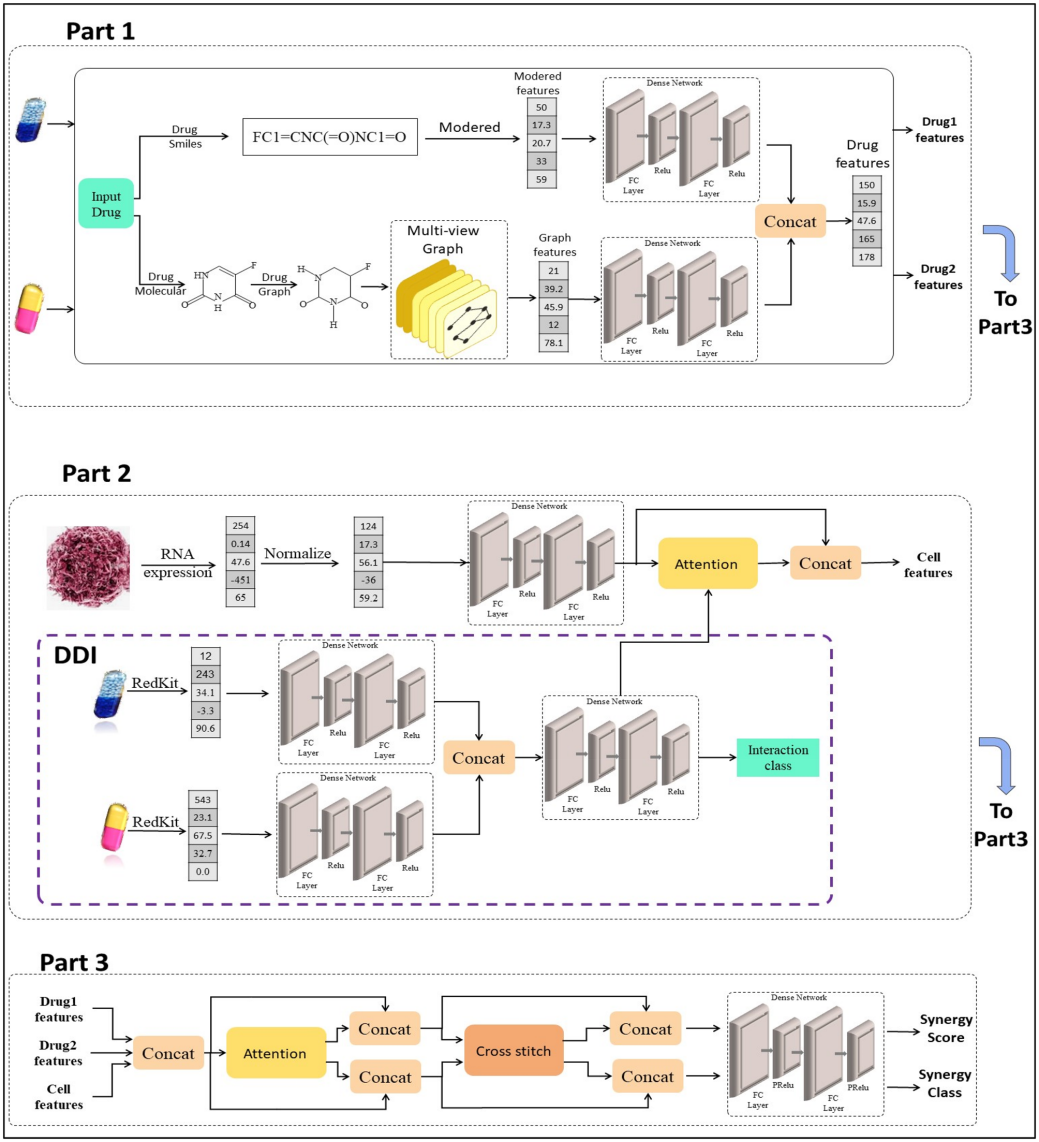
The structure of the proposed MultSyn model. The first part learned the drug features which the upper part extracts the chemical features from smiles and the lower part extract the drug graph features from molecular structure. The theses two features are concatenated to output the drug features. the second part, learned the cell line feature from the RNA-expression and the impact of drug-drug interaction features. In the third part the drugs feature and cell line feature are concatenated are fed to an attention and cross-stitch mechanism to optimized the target tasks.

The proposed multi-task model focuses on predicting the synergistic effects of drug combinations. The model generates both a synergy score and a synergy class label indicating whether the combination is synergistic or antagonistic. As shown in Fig. 3, the model can be divided into three main parts. The first part deals with the features of the individual drugs, the second part handles the features of the cancer cell lines, and the third part combines the drug and cell line features to produce the synergy score and class label simultaneously. The three subsequent subsections will discuss these three parts respectively in detail, highlighting their respective roles and functionalities.

### Drug feature representation

Two methods are employed to extract drug features: chemical structure-based extraction and graph embedding-based extraction.

In the chemical structure-based method, the SMILE representations of drugs are obtained from the PubChem website. These representations are then transformed into molecular feature vectors using the chemical informatics package from DeepChem^9^, specifically utilizing the "Mordred" features^10^. This process yields an array consisting of 1613 numeric features across 43 different categories, providing a one-dimensional molecular description for each drug. Non-numerical attributes and features with zero variance are removed through pre-processing, resulting in 394 descriptive features for each drug. The resulting features are then normalized using the tanh-norm method.

Next, the features are fed into a subnetwork that includes three fully connected layers, interconnected by an activation function and a dropout layer. The Rectified Linear Unit (ReLU) activation function is applied after each fully connected layer. A dropout rate of 0.2 is applied after the first and second fully connected layers, while the final fully connected layer does not employ dropout. To prevent overfitting, each fully connected layer undergoes a regularization technique applied to the weight and output of the layer.

**Figure 4:**
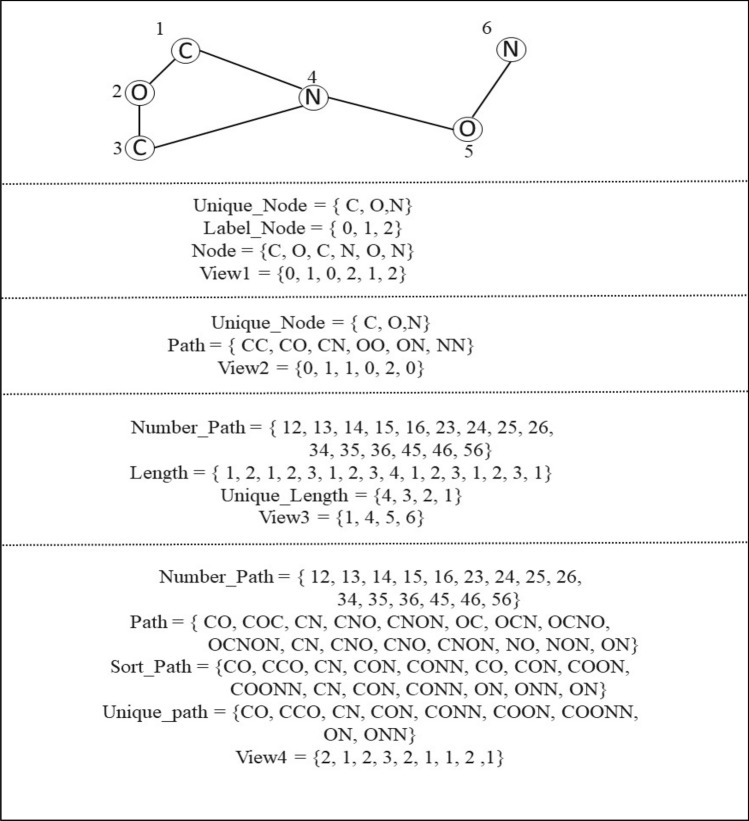
An example of multi-view graph embedding. Each section executes each view of the above graph results in the four views of graph.

In the graph embedding-based method, the SMILE representations of drugs are transformed into graphs, where each atom represents a node and the chemical bonds between atoms represent edges. Specifically, we improved a multi-view graph embedding technique to extract four views of the graphs. For each view, we initialize the output vector as a fixed-length vector filled with zeros to ensure consistency across views. Then, the vector is modified based on the output of each view. Finally, the four view vectors are concatenated into a single vector. Figure 4 shows an example for the four views. These views are discussed below in more detail:

I. The first perspective is first proposed in this paper is focused on the labeling of nodes in a graph. Initially, the unique nodes in the graph are identified and assigned a distinct numerical value in order to differentiate them. Then, the view is proposed as a vector that represents the corresponding numeric value of each node in the graph.
II. In the second view, the information from labels associated with each edge in the graph is utilized. First, the unique nodes in graph is utilized. Then, all paths between each node and others, including self-paths (loops) are considered. The occurrence number of each path is calculated, resulting in the final vector that contains the occurrence numbers of all paths. The application of this view to extract features is done as proposed in^11^.
III. The third view focuses on extracting the density of the neighborhood of each atom by examining the shortest path length between atoms. All possible paths between all nodes are considered and the length for each path is calculated. The occurrence number of each unique path length is output in the final view vector. This view was also proposed in^11^.
IV. In the fourth view, the information from labels in all possible paths between all nodes in the graph is utilized. Similar to the second view, we consider all possible paths, but instead of counting the occurrence number of each unique length, the occurrence number of each unique label path is counted. The resulting view vector is constructed based on this information. This view feature extraction was proposed in^12^.

The four views, comprising the multi-view graph embedding, are concatenated into a single vector, which is then normalized using the tanh-norm method. This resultant vector is then fed into a fully connected subnetwork like the structure fully connected subnetwork. The output vector from the fully connected structure subnetwork is concatenated with the output vector from the fully connected multi-view graph network. The resulting vector represents the final drug features.

### Cell line features representation

The extraction of cell line features can be divided into two sections. The first section deals with the initial representation of cell line features, while the second section focuses on how the interaction features of drugs can affect the cell line features.

In the first section, the cell line features are represented using gene expression dataset, which typically consists of over 50,000 gene features per cell line. To address the challenge of high dimensionality, we leverage the LINCS project^13^. This project identifies a subset of crucial genes, known as the 'Landmark gene set', that capture approximately 80% of cell line characteristics based on connectivity map data. These genes are consisting of 1000 carefully selected genes.

To obtain the initial cell line vector, the genes that intersect between the gene expression data and the Landmark gene set are selected. This results in the selection of 875 genes that effectively represent the cell line. These genes are then normalized using the tanh-norm method and fed into a fully connected subnetwork to extract the cell line features.

In the second section, the aim is to explore how drug interaction features affect cell line features through a drug-drug interaction (DDI) network. Initially, drugs are represented using a chemical structure-based extraction method. Two parallel fully connected subnetworks are then trained, each dedicated to one drug. The outputs from these subnetworks are concatenated into a single vector, which is subsequently fed into another fully connected subnetwork to generate the final class label. The DDI network is trained using the DrugBank drug-drug interaction dataset^14,15^. This dataset comprises 1706 unique drugs, resulting in 191,808 drug interaction pairs across 86 interaction class types. The dataset is split into a 9:1 ratio for training and validation data. Table 1 presents the validation metrics for the drug-drug interaction dataset.

**Table 1:** The evaluation metrics of DDI model.

Metrics	Values
Accuracy	0.969
Precision	0.970
Recall	0.969
Kappa	0.966
F1-score	0.971

From the learned DDI network, the drug interaction features are extracted from the penultimate layer of the fully connected subnetwork. These features are then normalized using the tanh-norm method and fed into a fully connected network to obtain the final representation of drug interaction features.

To integrate the cell line features and drug interaction features, an attention mechanism discussed in the previous section (“Attention mechanism”) is applied. The cell line features serve as the query for the attention mechanism, while the drug interaction features act as the key and value inputs. The output of the attention mechanism represents the updated cell line features, which are influenced by the weights assigned to the drug interaction features.

### Multi-output tasks

This section focuses on combining the drug and cell line features to generate the synergy score and synergy class label simultaneously.

To begin, the drug features and cell line features are concatenated and passed through an attention mechanism. As discussed previously, the attention mechanism enables different weighting of the combined features for each task. Consequently, the output of the attention mechanism produces two weighted feature representations, one for each task. These outputs are then concatenated with the respective input attentions for each task.

The resulting combined features are subsequently inputted into a cross-stitch mechanism described in previous “Cross-stitch mechanism”. This network learns the relationship between the synergy score and synergy class label tasks. The cross-stitch network produces two outputs, representing the feature representations for the two task relationships. Additionally, the outputs of the cross-stitch network are concatenated with the inputs of the cross-stitch network for each task.

Finally, the two sets of feature representations are passed through separate fully connected networks, with one network dedicated to each task. These networks output the synergy score and synergy class label, respectively.

## Experimental results

In this section, we evaluate the performance of the MutliSyn using one of the O'Neil challenging datasets^16^. We begin by providing an overview of the dataset and discuss the evaluation metrics in the first and second subsections respectively. Next, we present the model parameter setting and discuss the experimental results of the MutliSyn on the target dataset in the third and fourth subsections respectively.

### Dataset characteristics

The drug combination dataset used in this study is derived from the O'Neil dataset, a large-scale published cancer screening dataset, and serves as a benchmark set. The dataset consists of information on drug combinations, including the names of the two drugs being combined and the specific cancer cell line targeted for treatment. It comprises 38 unique drugs that are combined to form a total of 23,052 drug combinations. These combinations are tested on 39 different cancer cell lines, covering seven cancer tissue types: skin, ovary, lung, large intestine, breast, prostate, and pleura.

To determine the impact of a pharmacological combination (synergistic or antagonistic), the Loewe Additivity score^17^ is calculated. This score is derived from a 4 × 4 dose–response matrix and assumes no interaction between a drug and itself. The range of synergy scores in the O'Neil dataset spans from −326.464 to 179.1233. It's worth noting that the dataset may contain multiple evaluations of the same drug combination in the initial data. To address this, the average of the replicate scores is used as the target synergy score for each unique drug pair and cell line combination resulting in 22,737 samples.

For the classification task, drug synergy is treated as a binary classification problem. Drug combinations with a synergy score greater than 30 are classified as synergistic, while combinations with a score less than 0 are considered antagonistic. Combinations with scores between 0 and 30 are excluded from the training set as they are considered additive combinations, resulting in a balanced sample distribution for the classification task. However, in this paper, removing samples in the classification task also leads to the removal of the same samples in the regression task. To mitigate this issue, a three-class labeling approach is used for the classification model, where synergy scores above 30 are assigned to the synergetic class, scores below 0 are assigned to the antagonistic class, and the remaining scores fall into the additive class. This introduces an imbalance in the sample distribution across the three classes, which may impact the classification training. The results of both the synergetic and antagonistic classes are reported and compared with other related works.

To ensure unbiased evaluation, the dataset is randomly split into five cross-fold validations using four distinct methods. The first method guarantees that each drug-drug combination pair appears in only one-fold. The second method ensures that each cell line is exclusively assigned to one-fold. The third and fourth methods ensure that each fold contains unique first drugs and second drugs, respectively. Additionally, the dataset is concatenated to create a reversed order for input drug pairs.

During each cross-fold validation, one-fold serves as the testing dataset, while the remaining four folds constitute the training dataset for the model. Subsequently, the mean synergy score and class labels prediction scores are computed across all five training runs and reported as the final results.

### Evaluation metrics

In evaluating the MutliSyn, various regression metrics are utilized. The first metric is the mean squared error (${\text{MSE}}$), which quantifies the squared difference between the predicted and actual scores. Additionally, the root mean squared error (${\text{RMSE}}$) is computed, representing the square root of the ${\text{MSE}}$. Furthermore, the 95% confidence interval for the MSE is calculated and proposed. Another important metric is the Pearson correlation coefficient (${{\text{CC}}}_{{\text{P}}}$), which evaluates the consistency between the predicted scores and the actual scores. Given the adoption of a five folds cross-validation approach, the mean and standard deviation of each evaluation metric are computed across the five folds to ensure the robustness and reliability of the results.

When evaluating the classification task, several metrics are employed to assess the performance of the MutliSyn. Firstly, accuracy is utilized to measure the proportion of correct predictions made by the model. However, due to the presence of imbalanced data in the test dataset, where negative samples dominate, accuracy alone may not provide a comprehensive understanding of the classifier's performance. Hence, precision is employed to evaluate how accurately the model predicts the synergetic class. Additionally, Cohen's Kappa is employed, which compares the classifier's performance to that of a classifier that randomly guesses based on the class frequencies, providing a measure of how much better the model is performing.

Moreover, two essential metrics are employed, particularly effective for imbalanced classification tasks with limited samples in the minority class. These metrics are the receiver operating characteristic curve (ROC-AUC) and the area under the precision-recall curve (PR-AUC). The ROC-AUC measures the classifier's ability to distinguish between positive and negative samples across various threshold settings, while the PR-AUC focuses on the precision-recall trade-off, which is especially important when dealing with imbalanced data.

### Global model setting

To fully define the MutliSyn, several global parameters are specified in Table 2. The hidden units for the fully connected subnetwork handling drug features are set to [258, 128] for the multi-view graph embedding and [1024, 512,256] for the chemical structure-based features. The hidden units for the cell line fully connected subnetwork are defined as [512, 265, 128]. For the prediction subnetwork, the hidden units are set as [128, 64] for both tasks. Additionally, the attention mechanism employed in the model has its output size set to match the input size, and the number of attention heads is set to 4.

**Table 2:** Hyperparameter settings of MutliSyn model.

	Hyperparameters
Hidden units of drugs subnetworks	[1024, 512, 256]
Hidden units of cell subnetwork	[512, 256, 128]
Hidden units of DDI subnetwork	[1024, 512, 256]
Hidden units of graph subnetwork	[258, 128]
Hidden units of prediction subnetworks	[128, 64]
Dropout rate	0.2
Head attention	4
Optimizer settings	Lr = 0.00001, weight_decay = 0.025, epoch = 1000, and batch_size = 64

During training, the model adopts a learning rate of 0.00001, a batch size of 64, and runs for 1000 iterations. A dropout rate of 0.2 is also applied. The model optimization is performed using the AdamW optimizer^18^, which is a variant of the Adam optimizer incorporating weight decay into the optimization process. Weight decay is a regularization technique that penalizes large weights during training, leading to simpler and more generalizable models. This regularization helps prevent overfitting and enhances the model's ability to predict new data. The weight decay value employed in this paper is 0.025.

## Results and discussion

Table 3 provides a summary of the experimental results, comparing the MutliSyn with others on the regression task with leaving each drug-drug combination pair appears in one-fold. The table shows the performance of the models in predicting synergy scores using various regression evaluation metrics. Notably, the MutliSyn outperforms the other methods, achieving the lowest ${\text{MSE}}$ and ${\text{RMSE}}$, and the highest ${{\text{CC}}}_{{\text{P}}}$.

**Table 3:** Comparison of synergy score prediction results with other methods with leaving drugs combination out.

Method	MSE	RMSE	Confidence interval	${{\text{CC}}}_{{\text{P}}}$
MutliSyn	219.14 ± 39.59	14.75 ± 1.28	[170.00, 268.29]	0.76 ± 0.02
PRODeepSyn	229.49 ± 42.81	15.09 ± 1.37	[176.34,282.64]	0.75 ± 0.02
AudnnSynergy	241.12 ± 43.52	15.46 ± 1.44	[187.09,295.15]	0.74 ± 0.03
DeepSynergy	255.49	15.91 ± 1.56	[239.93, 271.06]	0.73 ± 0.04
XGBoost	310.50 ± 50.57	17.56 ± 1.42	[247.73, 373.28]	0.64 ± 0.02
Elastic-Net	425.50 ± 54.70	20.59 ± 1.31	[357.59, 493.41]	0.44 ± 0.02

The MutliSyn achieves an ${\text{MSE}}$ of 219.14, accompanied by a confidence interval ranging from 170.00 to 268.29. In comparison to PRODeepSyn, AudnnSynergy, and DeepSynergy, the MutliSyn demonstrates superior performance by achieving lower ${\text{MSE}}$ values, with improvements of −10.35, −21.98, and −36.35, respectively. These results indicate that the MutliSyn consistently delivers more accurate predictions, with significantly reduced prediction errors compared to the existing models.

Additionally, the MutliSyn exhibits an enhancement of 1.0% in terms of ${{\text{CC}}}_{{\text{P}}}$ compared to PRODeepSyn, 2.0% compared to AudnnSynergy, and 3.0% compared to DeepSynergy. This improvement emphasizes the effectiveness of the MutliSyn in accurately predicting synergy scores, effectively capturing the intricate relationship between drug combinations and their synergistic effects.

MutliSyn exhibits substantial enhancements across all prediction metrics for drug synergy when compared to machine learning algorithms, specifically Elastic-Net and XGBoost.

In Table 4, the prediction of synergy score depends on leaving cell line out. MutliSyn exhibits the ${\text{MSE}}$ and ${\text{RMSE}}$ among all methods, with values of 405.74 ± 104.32 and 19.96 ± 2.73, respectively. These metrics indicate the model's exceptional predictive accuracy and precision. Furthermore, MutliSyn demonstrates acceptable ${{\text{CC}}}_{{\text{P}}}$ of 0.50 ± 0.07, showcasing its robust ability to capture underlying patterns in the data and align well with ground truth labels. Notably, while PRODeepSyn achieves a slightly higher ${{\text{CC}}}_{{\text{P}}}$, the MutliSyn closely follows, positioning it as a competitive and promising model for synergy prediction. This places MutliSyn as the second-highest performer.

**Table 4:** Comparison of synergy score prediction results with other methods with leaving cell line out.

Method	MSE	RMSE	Confidence interval	${{\text{CC}}}_{{\text{P}}}$
MutliSyn	405.74 ± 104.32	19.96 ± 2.73	[276.23, 535.25]	0.50 ± 0.07
PRODeepSyn	440.91 ± 113.07	20.80 ± 2.85	[300.53, 581.28]	**0.52 ± 0.07**
AudnnSynergy	453.28 ± 111.21	21.11 ± 2.78	[315.21, 591.34]	0.41 ± 0.05
DeepSynergy	435.82 ± 87.51	20.77 ± 2.15	[327.18, 544.45]	0.43 ± 0.05
XGBoost	460.46 ± 108.04	21.31 ± 2.49	[326.33, 594.58]	0.43 ± 0.06
Elastic-Net	418.02 ± 105.76	20.27 ± 2.65	[286.73, 549.32]	0.42 ± 0.06

In Tables 5 and 6, MutliSyn demonstrates outstanding performance, yielding the lowest ${\text{MSE}}$ and ${\text{RMSE}}$ values and highest ${{\text{CC}}}_{{\text{P}}}$ in comparison to other models, including PRODeepSyn, AudnnSynergy, DeepSynergy, XGBoost, and Elastic-Net.

**Table 5:** Comparison of synergy score prediction results with other methods with leaving first drug out.

Method	MSE	RMSE	Confidence interval	${{\text{CC}}}_{{\text{P}}}$
MutliSyn	279.39 ± 47.16	16.66 ± 1.41	[220.84, 337.93]	0.68 ± 0.06
PRODeepSyn	317.36 ± 42.94	17.77 ± 1.25	[264.06, 370.66]	0.63 ± 0.11
AudnnSynergy	353.62 ± 50.67	18.76 ± 1.36	[290.71, 416.52]	0.57 ± 0.08
DeepSynergy	496.32 ± 94.83	22.17 ± 2.18	[378.59, 614.05]	0.39 ± 0.04
XGBoost	365.08 ± 57.56	19.05 ± 1.53	[293.61, 436.54]	0.56 ± 0.06
Elastic-Net	448.37 ± 55.91	21.13 ± 1.35	[378.96, 517.78]	0.40 ± 0.06

**Table 6:** Comparison of synergy score prediction results with other methods with leaving second drug out.

Method	MSE	RMSE	Confidence interval	${{\text{CC}}}_{{\text{P}}}$
MutliSyn	291.11 ± 50.62	17.00 ± 1.41	[228.27, 353.96]	0.65 ± 0.09
PRODeepSyn	302.91 ± 73.50	17.29 ± 2.00	[211.67, 394.15]	0.64 ± 0.09
AudnnSynergy	372.52 ± 37.15	19.28 ± 0.95	[326.40, 418.63]	0.53 ± 0.08
DeepSynergy	512.53 ± 56.50	22.60 ± 1.28	[442.39, 582.67]	0.33 ± 0.03
XGBoost	364.85 ± 42.59	19.07 ± 1.10	[311.97, 417.73]	0.52 ± 0.09
Elastic-Net	453.59 ± 42.02	21.28 ± 0.98	[401.42, 505.76]	0.34 ± 0.12

When considering the classification of synergistic drug combinations, Table 7 presents the performance model for predicting the synergy class label in comparison to other methods with drug-drug combination pair method. Although the MutliSyn does not achieve the highest accuracy score among the other methods, this is due to the fact that it does not eliminate the additive class, as discussed in “Dataset characteristics”. Furthermore, accuracy alone cannot be considered a fair metric for imbalanced classification predictions, as previously mentioned.

**Table 7:** Comparison of synergy class labels prediction results with other methods with leaving drug combination pair out.

Method	Accuracy	ROC-AUC	PR-AUC	Precision	Kappa
MutliSyn	0.90 ± 0.02	0.95 ± 0.02	0.85 ± 0.03	0.93 ± 0.01	0.61 ± 0.06
PRODeepSyn	0.93 ± 0.01	0.90 ± 0.03	0.63 ± 0.05	0.72 ± 0.06	0.51 ± 0.03
AudnnSynergy	0.93 ± 0.01	0.91 ± 0.02	0.63 ± 0.06	0.72 ± 0.06	0.51 ± 0.04
DeepSynergy	0.92 ± 0.03	0.90 ± 0.03	0.59 ± 0.06	0.56 ± 0.11	0.51 ± 0.04
XGBoost	0.89 ± 0.02	0.85 ± 0.03	0.65 ± 0.05	0.70 ± 0.03	0.66 ± 0.04
Elastic-Net	0.76 ± 0.03	0.81 ± 0.04	0.47 ± 0.06	0.42 ± 0.05	0.41 ± 0.05

However, when evaluating other metrics such as Precision, ROC-AUC, and PR-AUC, the MutliSyn outperforms all related methods. While MutliSyn may not achieve the highest Kappa metric, it consistently demonstrates substantial agreement. Consequently, the MutliSyn exhibits exceptional performance in the task of synergistic drug combination classification with new drug-drug combination pairs.

Table 8 shows the classification performance of MutliSyn compared to other models with leaving cell line out method. MutliSyn achieves a high accuracy, Precision, and PR-AUC compared to other models, including PRODeepSyn, AudnnSynergy, DeepSynergy, XGBoost, and Elastic-Net. However, the variations observed in Kappa and ROC-AUC metrics for MutliSyn across five folds highlight potential challenges in maintaining consistent performance, particularly in the context of imbalanced classes. Specially, MultiSyn has been trained with additive class as discussed in “Dataset characteristics” which impacts the classification results.

**Table 8:** Comparison of synergy class labels prediction results with other methods with leaving cancer cell line out.

Method	Accuracy	ROC-AUC	PR-AUC	Precision	Kappa
MutliSyn	0.85 ± 0.04	0.80 ± 0.19	0.62 ± 0.23	0.70 ± 0.29	0.38 ± 0.21
PRODeepSyn	0.85 ± 0.06	0.83 ± 0.15	0.62 ± 0.23	0.62 ± 0.26	0.47 ± 0.26
AudnnSynergy	0.84 ± 0.03	0.81 ± 0.14	0.57 ± 0.21	0.60 ± 0.29	0.35 ± 0.22
DeepSynergy	0.82 ± 0.08	0.79 ± 0.16	0.53 ± 0.19	0.52 ± 0.19	0.47 ± 0.25
XGBoost	0.86 ± 0.07	0.78 ± 0.15	0.53 ± 0.20	0.60 ± 0.24	0.53 ± 0.28
Elastic-Net	0.82 ± 0.08	0.79 ± 0.15	0.49 ± 0.17	0.51 ± 0.1	0.48 ± 0.25

In Tables 9 and 10, MutliSyn achieves the highest ROC-AUC, PR-AUC, and Precision among the compared models, indicating its overall robustness. While MutliSyn still maintains good accuracy with the second highest accuracy value and an acceptable Kappa.

**Table 9:** Comparison of synergy class labels prediction results with other methods with leaving first drug out.

Method	Accuracy	ROC-AUC	PR-AUC	Precision	Kappa
MutliSyn	0.89 ± 0.03	0.93 ± 0.02	0.80 ± 0.09	0.91 ± 0.05	0.54 ± 0.08
PRODeepSyn	0.90 ± 0.01	0.92 ± 0.02	0.79 ± 0.05	0.80 ± 0.08	0.66 ± 0.05
AudnnSynergy	0.87 ± 0.01	0.84 ± 0.06	0.62 ± 0.16	0.74 ± 0.08	0.42 ± 0.18
DeepSynergy	0.78 ± 0.04	0.82 ± 0.03	0.54 ± 0.09	0.47 ± 0.13	0.43 ± 0.10
XGBoost	0.87 ± 0.02	0.81 ± 0.04	0.57 ± 0.09	0.67 ± 0.10	0.59 ± 0.06
Elastic-Net	0.76 ± 0.04	0.81 ± 0.02	0.47 ± 0.08	0.44 ± 0.09	0.42 ± 0.06

**Table 10:** Comparison of synergy class labels prediction results with other methods with leaving second drug out.

Method	Accuracy	ROC-AUC	PR-AUC	Precision	Kappa
MutliSyn	0.88 ± 0.03	0.90 ± 0.04	0.73 ± 0.16	0.88 ± 0.09	0.47 ± 0.11
PRODeepSyn	0.89 ± 0.01	0.90 ± 0.04	0.71 ± 0.17	0.71 ± 0.16	0.50 ± 0.11
AudnnSynergy	0.86 ± 0.03	0.84 ± 0.07	0.60 ± 0.20	0.67 ± 0.15	0.42 ± 0.13
DeepSynergy	0.71 ± 0.08	0.71 ± 0.07	0.37 ± 0.12	0.39 ± 0.19	0.21 ± 0.14
XGBoost	0.87 ± 0.02	0.76 ± 0.07	0.51 ± 0.17	0.62 ± 0.15	0.52 ± 0.14
Elastic-Net	0.74 ± 0.07	0.74 ± 0.12	0.40 ± 0.20	0.37 ± 0.17	0.33 ± 0.19

**Figure 5:**
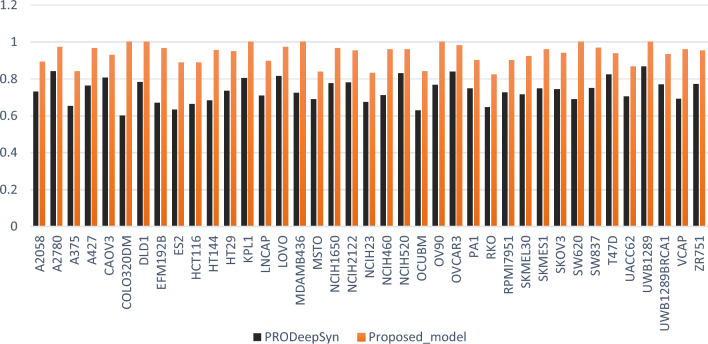
Precision Score Performance comparison between the proposed model and PRODeepSyn.

Furthermore, to visualize the results of the MutliSyn in comparison to other methods, Fig. 5 presents the Precision values for each cancer cell line, comparing the MutliSyn to PRODeepSyn. As depicted in Fig. 5, the MutliSyn demonstrates a significant improvement in precision across all cancer cell lines.

**Figure 6:**
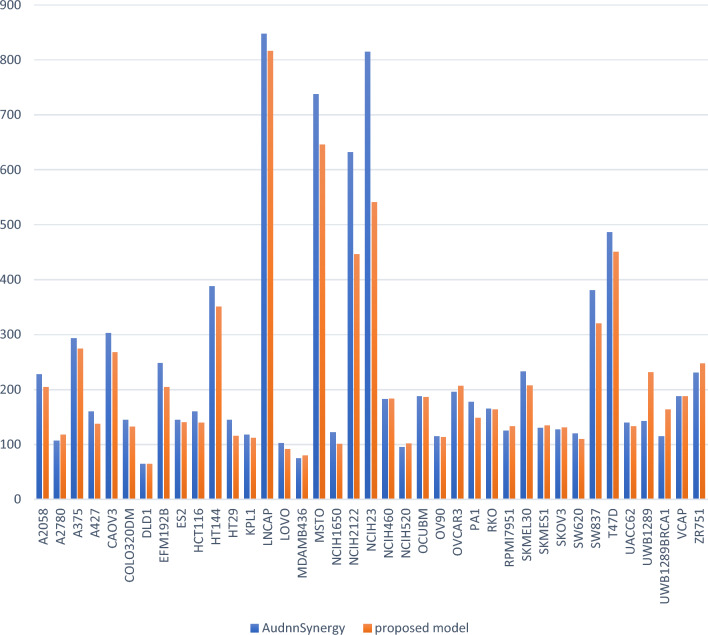
MSE Score Performance comparison between the proposed model and AudnnSynergy.

Moreover, Fig. 6 displays a comparison between the MutliSyn and AudnnSynergy in terms of ${\text{MSE}}$ across all cancer cell lines. The MutliSyn achieves a reduction in ${\text{MSE}}$ for 21 out of 39 cancer cell lines, while it performs almost similarly to AudnnSynergy for 10 out of 39 cell lines. However, AudnnSynergy outperforms the MutliSyn in terms of ${\text{MSE}}$ for 8 out of 39 cell lines. Notably, the greatest improvement observed with the MutliSyn, compared to AudnnSynergy, is in the 'NCH23' cell line, resulting in a reduction of almost 270 points in MSE. Conversely, AudnnSynergy exhibits the highest improvement compared to the MutliSyn in the ‘UWB1289’ cell line, with a reduction of almost 89 points in MSE. AudnnSynergy outperforms our model significantly, particularly for both 'UWB1289' and 'UWB1289BRCA1.' This is noteworthy because our model employed the same gene expression data for 'UWB1289BRCA1' as for 'UWB1289,' recognizing the latter as a variant of the former.

To delve deeper into the analysis, we conducted an ablation study on various MutliSyn model configurations by selectively removing specific components of its architecture. The study focuses on the regression values of synergy scores for drug-drug combination pairs. The results of this ablation study are presented in Table 11. In the first section, we remove the cross-stitch mechanism from the MutliSyn model, connecting the output of the concatenated attention mechanism directly to the prediction network. Moving to the second section, referred to as feature-attention, we eliminate the attention mechanism responsible for customizing the concatenated drugs and cell line features. Instead, the concatenated features are linked to two fully connected layers, serving as inputs to the cross-stitch mechanism. The third section, interaction-attention, involves the removal of the attention mechanism between cell line features and DDI features. Consequently, DDI features are excluded, and cell line features are directly fed into the concatenated features stage. Lastly, the unenhanced graph views involve utilizing only views numbered {2, 3, 4}, excluding view number {1} proposed in that paper.

**Table 11:** The comparison results of the MultiSyn ablation study at synergy score.

Method	MSE	RMSE	Confidence interval	${{\text{CC}}}_{{\text{P}}}$
Cross-stitch	226.69 ± 44.73	14.99 ± 1.42	[171.17, 282.22]	0.75 ± 0.03
Feature-attention	225.99 ± 44.69	14.97 ± 1.42	[170.51, 281.48]	0.75 ± 0.03
Interaction-attention	221.45 ± 41.91	14.85 ± 1.35	[170.43, 272.48]	0.76 ± 0.03
Unenhanced graph views	221.65 ± 43.49	14.82 ± 1.39	[167.65, 275.64]	0.76 ± 0.03
MutliSyn	219.14 ± 39.59	14.75 ± 1.28	[170.00, 268.29]	0.76 ± 0.02

Finally, Table 12 displays the highest predicted synergy scores for drugs combination not presented in O’Neil dataset, along with the corresponding actual synergy scores for the MutliSyn*.

**Table 12:** The top predicted synergistic drugs combination for each cancer cell.

Cell_line	Drug1	Drug2	Predict	Actual
A2058	Etoposide	Gemcitabine	0.17	−6.44
A375	Etoposide	Gemcitabine	14.77	18.88
RPMI7951	Etoposide	Gemcitabine	−6.03	−10.64
SKMEL30	Etoposide	Gemcitabine	2.67	−0.93
UACC62	Etoposide	Gemcitabine	−12.08	−17.98
HCT116	Methotrexate	Cyclophosphamide	−18.67	−25.86
HT29	Methotrexate	Cyclophosphamide	−5.55	−4.08
NCIH23	Methotrexate	Cyclophosphamide	−23.98	−33.92
OVCAR3	Methotrexate	Cyclophosphamide	−31.69	−49.97
UACC62	Methotrexate	Cyclophosphamide	−38.75	−47.24
HCT116	Paclitaxel	Cyclophosphamide	−19.36	−26.66
HT29	Paclitaxel	Cyclophosphamide	−41.09	−49.13
NCIH23	Paclitaxel	Cyclophosphamide	−25.05	−31.72
OVCAR3	Paclitaxel	Cyclophosphamide	−35.45	−53.34
UACC62	Paclitaxel	Cyclophosphamide	−57.27	−74.85
HCT116	Paclitaxel	Methotrexate	−26.53	−28.2
HT29	Paclitaxel	Methotrexate	−29.12	−35.35
NCIH23	Paclitaxel	Methotrexate	−14.93	−14.65
OVCAR3	Paclitaxel	Methotrexate	−6.45	−8.68
UACC62	Paclitaxel	Methotrexate	−23.85	−22.13

Based on the analysis conducted, the MutliSyn demonstrates efficacy in predicting target synergy scores and classes for drug combinations. It showcases minimal prediction errors, a strong correlation between actual and predicted scores, and outperforms the other compared methods. Moreover, the model utilizes a multi-task deep learning approach to simultaneously predict outputs, which contributes to its enhanced performance.

## Conclusion

In this paper, a novel multi-task deep learning model has been proposed for the simultaneous prediction of both the synergy score and synergy class label for drug combinations. The model incorporates two different representations of drug features: chemical structure-based features and multi-view graph features. These features are combined into a single feature vector to represent the drug features. Additionally, the gene expression of the cancer cell line is utilized as an initial feature of the cell line. Furthermore, drug-drug interaction features have been extracted and integrated into an attention mechanism along with the cell line features, enabling the model to learn the impact of drug-drug features on the cell line and generate new cell line features. The drug and cell line features are concatenated and processed through an attention mechanism, which optimizes the concatenated features into two distinct representations for the two target output tasks. These task representations are then inputted into a cross-stitch algorithm to facilitate knowledge transfer between the tasks. Finally, each task representation has passed through a fully connected subnetwork to generate the desired target outputs.

The MutliSyn has been evaluated using the O'Neil cancer dataset, which contains information on drug combinations and cell lines. The results of the synergy score prediction indicate that the model achieves low MSE and RMSE, as well as high ${{\text{CC}}}_{{\text{P}}}$. Moreover, for the synergy class label prediction, the model demonstrates high Precision, ROC-AUC, Kappa, and PR-AUC values when compared to other deep learning models.

## Data Availability

The datasets utilized in this study were sourced from the O'Neil dataset, which can be accessed at the following link: (https://github.com/samar-monem/synergistic-model/tree/1571f93ece23cb9499bf1be51f86f4df6f148470/data). To process the drug features, the SMILES (Simplified Molecular Input Line Entry System) representations of the drugs were extracted from the PubChem website (https://pubchem.ncbi.nlm.nih.gov/). These SMILES were then transformed into chemical-based features using the DeepChem chemical informatics package, which is freely available. More information about DeepChem can be found at (https://deepchem.readthedocs.io/en/latest/api_reference/featurizers.html]. Regarding the gene expression of the cell lines, the majority of the data was obtained from [https://depmap.org/portal/) and corresponds to a file named "CCLE_expression". However, for the 'OCCUBM' cell line, its gene expression data was sourced from (https://cellmodelpassports.sanger.ac.uk/passports/SIDM00241).

## Code availability

The code of the MutliSyn has been implemented and is accessible at (https://github.com/samar-monem/synergistic-model.git).
